# Supplementary material for: The number of cytokinesis nodes in mitotic fission yeast scales with cell size
Source: eLife. 2022 Sep 12;11:e76249. doi: 10.7554/eLife.76249 (PMC9467510; doi:10.7554/eLife.76249)
Supplement: Transparent reporting form [file elife-76249-transrepform1.pdf]

eLIFE

1st Floor
24 Hills Road
Cambridge CB2 1JP, UK

P 01223 855340
W elifesciences.org
T @elife

eLife's transparent reporting form

We encourage authors to provide detailed information *within their submission* to facilitate the interpretation and replication of experiments. Authors can upload supporting documentation to indicate the use of appropriate reporting guidelines for health-related research (see EQUATOR Network), life science research (see the BioSharing Information Resource), or the ARRIVE guidelines for reporting work involving animal research. Where applicable, authors should refer to any relevant reporting standards documents in this form.

If you have any questions, please consult our Journal Policies and/or contact us: editorial@elifesciences.org.

Sample-size estimation

- You should state whether an appropriate sample size was computed when the study was being designed
- You should state the statistical method of sample size computation and any required assumptions
- If no explicit power analysis was used, you should describe how you decided what sample (replicate) size (number) to use

Please outline where this information can be found within the submission (e.g., sections or figure legends), or explain why this information doesn't apply to your submission:

Each figure legend contains the number of samples, statistical tests, and results. Individual data points along with the average and SD or range are shown in figures wherever possible. Sample sizes were decided based on the previously published datasets with 6-21 cells per strain or conditions.

Replicates

- You should report how often each experiment was performed
- You should include a definition of biological versus technical replication
- The data obtained should be provided and sufficient information should be provided to indicate the number of independent biological and/or technical replicates
- If you encountered any outliers, you should describe how these were handled
- Criteria for exclusion/inclusion of data should be clearly stated
- High-throughput sequence data should be uploaded before submission, with a private link for reviewers provided (these are available from both GEO and ArrayExpress)

Please outline where this information can be found within the submission (e.g., sections or figure legends), or explain why this information doesn't apply to your submission:

Replicate values are present for all data in accompanying Figure legends Individual data points are shown in each figure and numbers are mentioned in legends for the complete data set. Average values with the SD or range are also shown as error bars in the figures or results sections without data exclusion.

eLife Sciences Publications, Ltd is a limited liability non-profit non-stock corporation incorporated in the State of Delaware, USA, with company number 5030732, and is registered in the UK with company number FC030576 and branch number BR015634 at the address 1st Floor, 24 Hills Road, Cambridge CB2 1JP | August 2014

Statistical reporting

- Statistical analysis methods should be described and justified
- Raw data should be presented in figures whenever informative to do so (typically when N per group is less than 10)
- For each experiment, you should identify the statistical tests used, exact values of N, definitions of center, methods of multiple test correction, and dispersion and precision measures (e.g., mean, median, SD, SEM, confidence intervals; and, for the major substantive results, a measure of effect size (e.g., Pearson's r, Cohen's d)
- Report exact p-values wherever possible alongside the summary statistics and 95% confidence intervals. These should be reported for all key questions and not only when the p-value is less than 0.05.

Please outline where this information can be found within the submission (e.g., sections or figure legends), or explain why this information doesn't apply to your submission:

Statistical analysis methods and their results are described in the figure legends
along with the precision measures, p-values, and exact N values, except in Figure
2D and Figure 3G. For Figure 2D and Figure 3G, Shapiro-Wilk W test (W ≥0.97) and Z score (<3.29) were used to show
that fluorescence intensities of single cytokinesis nodes are distributed
normally with a mean of 2300 and SD of 900 (AU). Individual data
points are shown in all the figures along with the average and SD or range.

(For large datasets, or papers with a very large number of statistical tests, you may upload a single table file with tests, Ns, etc., with reference to sections in the manuscript.)

Group allocation

- Indicate how samples were allocated into experimental groups (in the case of clinical studies, please specify allocation to treatment method); if randomization was used, please also state if restricted randomization was applied
- Indicate if masking was used during group allocation, data collection and/or data analysis

Please outline where this information can be found within the submission (e.g., sections or figure legends), or explain why this information doesn't apply to your submission:

Samples were allocated into the experimental groups based on genotype, the cell size, or growing conditions as described in the text, figures, and figure legends. randomization was not applied.

Additional data files ("source data")

- We encourage you to upload relevant additional data files, such as numerical data that are represented as a graph in a figure, or as a summary table
- Where provided, these should be in the most useful format, and they can be uploaded as "Source data" files linked to a main figure or table
- Include model definition files including the full list of parameters used
- Include code used for data analysis (e.g., R, MatLab)
- Avoid stating that data files are "available upon request"

Please indicate the figures or tables for which source data files have been provided:

All the data is provided in the manuscript. The genotypes of yeast strains used in this study are listed in Table 1 which is included in the Methods and Materials
sections. We have included the MATLAB codes for extracting single node intensity from broad band of nodes or contractile rings along with the cell size parameters such as length, diameter, surface area, and volume as Supplementary File - "Source Code1_20220805.zip". The MATLAB codes need ROI-set files from ImageJ containing x-y-z positions of individual nodes and raw two-coloured z-stack Airyscan images containing 40 z-slices. Supplementary figures, Figure 3-figure supplement 1 and Figure 3-figure supplement 2 are also included at the end of the manuscript.

eLife Sciences Publications, Ltd is a limited liability non-profit non-stock corporation incorporated in the State of Delaware, USA, with company number 5030732, and is registered in the UK with company number FC030576 and branch number BR015634 at the address 1st Floor, 24 Hills Road, Cambridge CB2 1JP | August 2014
